# Pheochromocytoma and Neurofibromatosis Type 1 in a Patient with Hypertension

**DOI:** 10.3889/oamjms.2015.130

**Published:** 2015-12-06

**Authors:** Julijana Petrovska, Biljana Gerasimovska Kitanovska, Stevka Bogdanovska, Svetlana Pavleska Kuzmanoska

**Affiliations:** 1 *Medical Faculty, Ss Cyril and Methodius University of Skopje, Skopje, Republic of Macedonia (Resident in gastroenterology)*; 2 *University Clinic of Nephrology, Medical Faculty, Ss Cyril and Methodius University of Skopje, Skopje, Republic of Macedonia*

**Keywords:** Pheochromocytoma, neurofibromatosis type 1, hypertension, antihypertensives, adrenalectomy

## Abstract

**BACKGROUND:**

Neurofibromatosis type 1 is an autosomal dominant condition that has a variety of clinical manifestations. Essential or secondary hypertension may be associated with neurofibromatosis. A rare finding is hypertension due to pheochromocytoma in patient with neurofibromatosis type 1.

**CASE REPORT:**

We present a case with a 7-year medical history of hypertension which was poorly controlled and with wide variations of blood pressure before the examination. Investigations did not reveal a secondary cause of hypertension. After the physical examination and establishing the diagnosis of neurofibromatosis, as well as the history of symptomes suggestive of catecholamine discharge, diagnostic procedures for pheochromocytoma were undertaken. Abdominal CT and MRI have proven the presence of a right adrenal tumor mass which was suspected to be a pheochromocytoma. Patient was preoperatively treated for two weeks with alpha and beta blokers and right adrenalectomy was performed. Perioperatively and on a longer term, blood pressure remained well controlled with less antihypertensive therapy. Diagnosis and management of pheochromocytoma in neurofibromatosis involves a dermatologist, endocrinologist, nephrologist and an urologist and requires a well-coordinated multidisciplinary approach.

**CONCLUSIONS:**

Pheochromocytoma, although a rare condition in patients with neurofibromatosis, may be a cause for uncontrolled hypertension, as well as other cardiovascular complications and the clinician should do all available clinical investigations to confirm it or exclude it on time.

## Introduction

Neurofibromatosis (NF) is a genetic disorder that involves multiple systems, and is known as neurocutaneous syndrome. Neurofibromatosis type 1 (NF1), also known as von Recklinghausen disease is an autosomal dominant condition [1]. The manifestations of NF1 result from a mutation in or deletion of the NF1gene with location at 17q11.2 [2]. The gene serves as tumour suppressor via its product called neurofibromin [3].

Neurofibromatosis type 1 may present with various cutaneous manifestations (cafe au lait patches, freckling on skin folds), iris Lisch nodules and neurofibromas. Less frequently it may involve central nervous tumors, malignant peripheral nerve sheath tumors, neurological, cognitive problems and ortopedic and gastrointestinal problems [4].

Pheochromocytoma occurs in 0.1%-5.7% of patients with NF-1. The incidence increases up to 20% among patients with hypertension [5]. Although rare, it may be the cause of uncontrolled hypertension as well as hypertension with wide variations. Diagnosis is a clinical one and high level of suspicion of the condition is needed to proceed with the clinical investigations.

We present a case with pheochromocytoma and neurofibromatosis type 1 with hypertension, who was successfully treated with unilateral laparoscopic adrenalectomy.

## Case description

The patient G.N, born in 1979, was referred to the University clinic of nephrology for clinical assessment for secondary hypertension. He had his first high blood pressure measurement 7 years ago, with maximum values for blood pressure up to 190/110 mmHg and presented with symptoms suggestive of catecholamine release. Investigations were started and therapy was given, but he had poor compliance due to blood pressure variability. He attended his general practicioner 2 monts before consulting to the clinic, and broad investigations for the etiology of the secondary hypertension were made.

Although regular antihypertensive treatment was continued, he had an elevation of the blood pressure (BP) up to 170/90 mmHg with clinical picture suggestive for catecholamine release. Supportive findings for secondary hypertension were those from abdominal ultrasound, renal ultrasound, abdominal CT and MRI, biochemistry, echocardiography, dermatological and endocrinological examinations. Past medical history included hyperthyroidism diagnosed 2 years ago and treated for 6 months, with no current treatment needed. His father had diabetes mellitus and no other significant diseases in the family were present.

**Figure 1:**
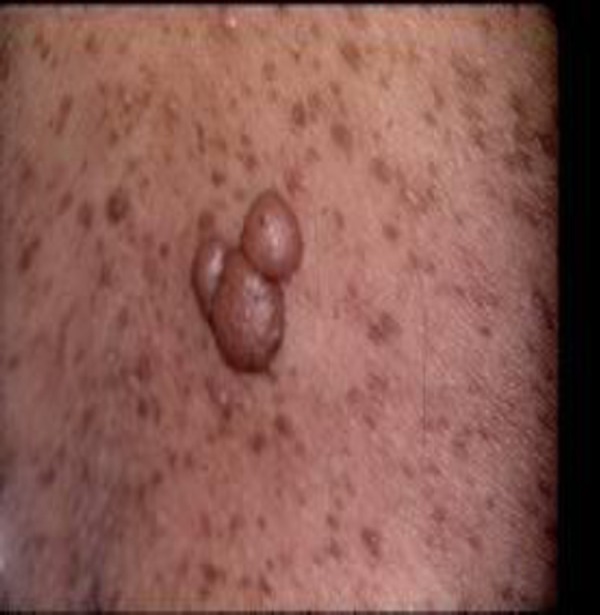
*Tumor mass in the right cervical area*.

**Figure 2:**
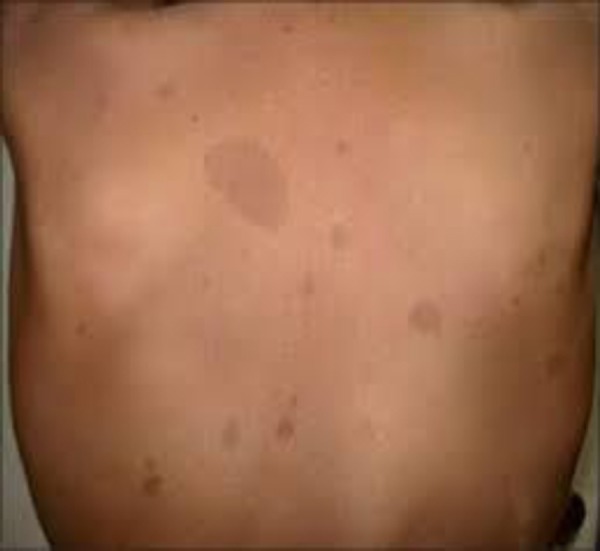
*Cafe-au-lait spots on the back*.

When the patient presented at the Clinic, his office blood pressure was 125/85 mmHg, and he was on treatment with a beta-blocker – Bisoprolol, an ACE inhibitor – Lisinopril, and Prazepam. A tumour mass was seen in the right cervical area and it was firm, limited and mobile, but the patient has done no further investigations for it (Fig. 1). Numerous discolorated patches were visible on the back, suggesting cafe-au-lait spots (Fig. 2).

Abdominal ultrasound showed a parenchymal, well-defined mass lesion size 51 × 53 mm seen at the area superior to the right upper kidney pole and below the right liver lobe, and adrenal gland origin could not be ruled out. Both kidneys had normal structure and location. Left adrenal gland was normal and in right adrenal gland, an oval, non-homogeneous tumour mass with dimensions – 54 × 63 mm was seen. Prostate was enlarged and homogeneous with size 33 × 32 × 30 mm.

**Figure 3:**
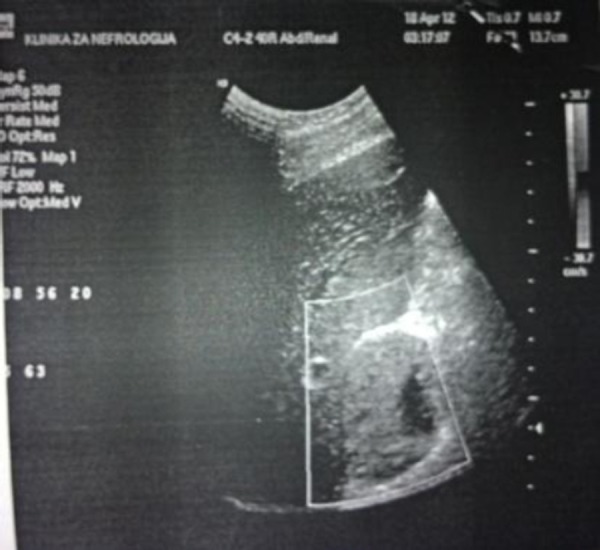
*Right suprarenal mass on abdominal ultrasound*.

Abdominal CT showed a well-defined, oval, soft tissue lesion, largest diameter of 6 cm, which suppresses the upper pole of the kidney seen in the right adrenal gland (Fig. 3). After application of contrast there was an intensive increment of attenuation with central hypoattenuation area of necrosis and increased attenuation persisted on the scan done 10 min later.

On MRI, cutaneous neurofibromas were seen over the left abdominal wall above the umbilicus. Liver, spleen, pancreas, left adrenal gland and both kidneys have normal morphology. In the right suprarenal area a well defined heterogeneous mass, size 6.0 × 5.5 cm, that suppress the upper pole of the right kidney was seen. MRI findings are consistent with Pheochromocytoma.

Laboratory findings revealed hyperglycemia but all other parameters were within normal (Table 1). Several other investigations were performed, considering the working diagnosis od neurofibromatosis and its possible variety of manifestations and consequences. Echocardiogram was normal. Ophthalmological findings were unremarkable. Dermatological exam diagnosed the condition as Neurofibromatosis type 1. Endocrinological consultation was consistent with Neurofibromatosis type 1, Right adrenal tumor, and Pheochromocytoma suspected, and surgical treatment was recommended.

**Table 1:** Laboratory findings of the patient at check-up at the Clinic

Erythrocytes:	5.0 (4.2-5.5 × 10^12^/l)

Platelets:	352 (140-340 × 10^9^/l)

Leucocytes:	9.8 (4-9 × 10^9^/l)

Hb	152 (140-180 g/l)

Neutrophils	0.71 (0.58-0.66)

Glucose :	6.5 (3.5-6.5 mmol/l)

Na-	146 (137-145 mmol/l)

K-	4.6 (3.8-5.5 mmol/l)

Ca-	2.5 (2.1-2.6 mmol/l)

Urea and creatinine –	within the normal reference range

Differential diagnosis includes metastatic lesions e.g. melanoma, pheochromocytoma (clinical and biochemical correlation is needed), adrenal carcinoma and neurofibroma.

Final diagnosis were secondary hypertension, right adrenal tumour and neurofibromatosis type 1 and two weeks prior to surgery, therapy with Prazosin 4 mg daily dose and in few days both Prazosin and Propranolol 120 mg daily dose were started.

**Figure 4:**
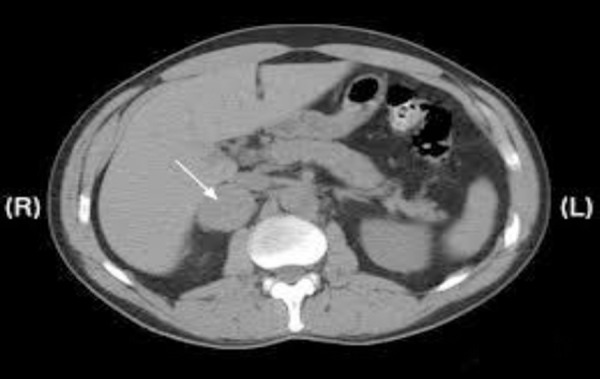
*Adrenal tumor on abdominal CT*.

The patient was admitted at University clinic of Urology and underwent laparoscopic removal of the tumour mass of the adrenal gland. Tumour consisted of mature feohromocytes (Fig. 4), with polygonal appearance and abundant basophilic granular cytoplasm. Immunophenotyping was consistent with neuroendocrine tumour markers CHR A (+) NSE (+).

**Figure 5:**
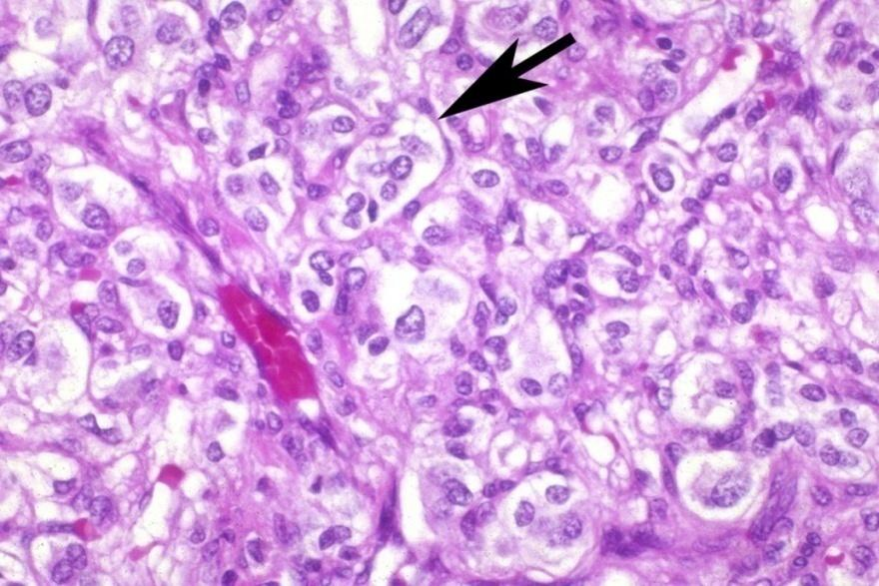
*Histological finding of the tumour - feochromocytes*.

After surgery, blood pressure was within normal range, and antihypertensive treatment was corrected. Two weeks after surgery the patient was in good general condition, blood pressure was in the normal range and antihypertensive therapy was stopped.

## Discussion

According to National Institutes of Health (NIH) Consensus Conference document developed in 1987 [1], neurofibromatosis can be diagnosed if two or more criteria are fulfilled: six or more cafèau-lait spots, two or more neurofibromas of any type, freckling in the axillary or the inguinal regions, optic glioma, two or more Lisch nodules; a typical osseous lesion or a first degree relative affected by NF1.

These diagnostic criteria are still valid and the diagnosis of patients with neurofibromatosis is a clinical one. Differential diagnosis should be made from other forms of neurofibromatosis, other conditions with cafe-au-lait patches and pigmented macules, overgrowth syndromes and other tumors confused with neurofibromatosis [4].

Our patient fulfilled the clinical criteria for diagnosis of neurofibromatosis type 1. Diagnosis of neurofibromatosis type 1 is often delayed and may attract medical attention when other symptoms and clinical problems arise. The condition is associated with vasculopathy which is 2.2 times more frequent in patients with neurofibromatosis aged 30–40-years. Vasculopathy may be associated to the protein neurofibromin, which is assocaited with lumen occlusion and hyperplasia of vessel intima. Often it is manifested with hypertension, which may be due to renal artery stenosis, coarctation of aorta, pheochromocytoma or may be essential [6].

Association between neurofibromatosis and pheochromocytoma was reported by Suzuki in 1910 [7]. It is an uncommon manifestation of neurofibromatosis and its incidence is reported as low. Yet, pheochromocytoma remains often, in up to 20% with neurofibromatosis type 1, undiagnosed when the level of clinical suspicion is low [8]. In 20-56% of patients with NF1 and hypertension, pheochromocytoma is diagnosed [9].

Our patient had a 7-year history of hypertension and when the variability of the blood pressure despite the use of antihypertensives became significant, the patient started clinical investigations. Catecholamine discharge which occurs in pheochromocytoma is not only a cause for variability of blood pressure, but it may cause myocardial injury and arrythmias [9].

The best diagnostic test for pheochromocytoma is serum metanephrine [10]. Computer tomography of the abdomen is the fundamental examination and MRI of the suprarenal glands has the highest sensitivity [11, 12]. Scintigraphy with MIBG is useful particularly when pheochromocytoma is not from adrenal origin. Unfortunately, the test for serum metanephrines is not available in our country. In our case, abdominal CT and MRI have correctly determined the location of the tumor.

Treatment of choice is adrenalectomy, which was performed in our patient after two weeks of preparation with medications. Beta blockers should be used only after successful alpha blockade. Our patient had an adequate preoperative and perioperative treatment and the long-term control of blood pressure was satisfactory. Cases report that pheochromocytoma is often an incidentaloma, with no clinical manifestations [13] and different outcomes are described, from well-controlled hypertension to acute myocardial infarction [14, 15].

Pheochromocytoma, although a rare condition in patients with neurofibromatosis, may be a cause for uncontrolled hypertension, as well as other cardiovascular complications and the clinician should do all available clinical investigations to confirm it or exclude it on time.

## References

[1] Group of authors (1988). Neurofibromatosis. Conference statement. National Institutes of Health Consensus Development Conference. Arch Neurol.

[2] Wallace MR, Marchuk DA, Anderson LB, Letcher R, Odeh HM, Saulino AM, Fountain JW, Bereton A, Nicholson J, Mitchell AL, Brownstein BH, Collins FS (1990). Type 1 neurofibromatosis gene;identification of a larger transcript disrupted in three NF1 patients. Science.

[3] Jett K, Friedman JM (2010). Clinical and genetic aspects of neurofibromatosis 1. Genetics in Medicine.

[4] Ferner RE, Huson SM, Thomas N, Moss C, Willshaw H, Evans G, Upadhyaya M, Towers R, Gleeson M, Steiger C, Kirby A (2007). Guidelines for the diagnosis and management of individuals with neurofibromatosis 1. J Med Genet.

[5] Walther MM, Herring J, Enquist E, Keiser HR, Linehan WM (1999). Von Recklinghausen’s disease and pheochromocytomas. J Urol.

[6] Rocchietti March M, De Palma C, L’Angiocola PD, Aliberti G (2008). Neurofibromatosis type I and hypertension: A case report. Recenti Prog Med.

[7] Suzuki S (1910). Ueber Zwei Tumoren aus Nebennierenmarkgewebe. Berlin Klein Wchnschr.

[8] Walther MM, Herring J, Enquist E, Keiser HR, Linehan WM (1999). Von Recklinghausen’s disease and pheochromocytomas. J Urol.

[9] Boulkina LS, Newton CA, Drake AJ (2007). Acute myocardial infarction attributable to adrenergic crises in patient with pheochromocytoma and neurofibromatosis 1. Endocr Pract.

[10] Darze ES, Von Sohsten RL (2004). Pheochromocytoma - induced segmental myocardial dysfunction mimicking an acute myocardial infarction in a patient with normal coronary arteries. Arq Bras Cardiol.

[11] (2006). PSH Working Group: Diagnostic and treatment guidelines for patients with chromaffinoma. Nadciś Tętn.

[12] Galati SJ, Said M, Gospin R, Babic N, Brown K, Geer EB, Kostakoglu L, Krakoff LR, Leibowitz AB, Mehta L, Muller S, Owen RP, Pertsemlidis DS, Wilck E, Xiao GQ, Levine AC, Inabnet WB (2015). The mount Sinai clinical pathway for the management of pheochromocytoma. Endocr Pract.

[13] Shinall MC, Solórzano CC (2014). Pheochromocytoma in Neurofibromatosis Type 1: When Should it Be Suspected?. Endocr Pract.

[14] Rocchietti March M, De Palma C, L’Angiocola PD, Aliberti G (2008). Neurofibromatosis type I and hypertension: A case report. Recenti Prog Med.

[15] Ahmed R, Darrat Y, Hamoudeh E, Elhamdani MO, Yaqub A (2014). Acute cardiomyopathy and multiorgan failure in a patient with pheochromocytoma and neurofibromatosis type 1. J Pak Med Assoc.

